# Real-time *in vivo* two-photon imaging study reveals decreased cerebro-vascular volume and increased blood-brain barrier permeability in chronically stressed mice

**DOI:** 10.1038/s41598-018-30875-y

**Published:** 2018-08-30

**Authors:** Sohee Lee, Bok-Man Kang, Jae Hwan Kim, Jiwoong Min, Hyung Seok Kim, Hyunwoo Ryu, Hyejin Park, Sungjun Bae, Daehwan Oh, Myunghwan Choi, Minah Suh

**Affiliations:** 1 Center for Neuroscience Imaging Research, Institute for Basic Science (IBS), Suwon, 16419 Republic of Korea; 2 Department of Biomedical Engineering, Sungkyunkwan University (SKKU), Suwon, 16419 Republic of Korea; 3 Biomedical Institute for Convergence at SKKU (BICS), Sungkyunkwan University (SKKU), Suwon, 16419 Republic of Korea; 4 Samsung Advanced Institute of Health Science and Technology (SAIHST), Sungkyunkwan University (SKKU), Suwon, 16419 Republic of Korea; 5 Department of Biological Science, Sungkyunkwan University (SKKU), Suwon, 16419 Republic of Korea

## Abstract

Chronic stress disrupts brain homeostasis and adversely affects the cerebro-vascular system. Even though the effects of chronic stress on brain system have been extensively studied, there are few *in vivo* dynamic studies on the effects of chronic stress on the cerebro-vascular system. In this study, the effects of chronic stress on cerebral vasculature and BBB permeability were studied using *in vivo* two-photon (2p) microscopic imaging with an injection of fluorescence-conjugated dextran. Our real-time 2p imaging results showed that chronic stress reduced the vessel diameter and reconstructed vascular volume, regardless of vessel type and branching order. BBB permeability was investigated with two different size of tracers. Stressed animals exhibited a greater BBB permeability to 40-kDa dextran, but not to 70-kDa dextran, which is suggestive of weakened vascular integrity following stress. Molecular analysis revealed significantly higher VEGFa mRNA expression and a reduction in claudin-5. In summary, chronic stress decreases the size of cerebral vessels and increases BBB permeability. These results may suggest that the sustained decrease in cerebro-vascular volume due to chronic stress leads to a hypoxic condition that causes molecular changes such as VEGF and claudin-5, which eventually impairs the function of BBB.

## Introduction

Chronic stress is known to affect our whole body and to have a negative effect on the cerebro-vascular system^1–3^. Adverse effects on the brain system of chronic stress include stroke, vascular dementia and cognitive dysfunction^4–6^. Especially, the detrimental effects of chronic stress on neuronal activation has been extensively studied over the past few decades, and decreased activation of the hippocampus, amygdala, pre-frontal cortex, in chronically stressed animals has been reported^7–10^. Because neuronal activation is tightly linked to brain hemodynamics, reduction in hemodynamics can be used as a measure of the decreased level of neuronal activation due to chronic stress. As evidence of this, decreased hemodynamic responses, such as decreased cerebral blood volume (CBV) and decreased BOLD fMRI signals, have been reported in chronically stressed animals and humans^1,11–13^. Recently, reduced cerebral blood volume (CBV) has been reported in chronically stressed rodents^12^. Chiba and colleagues reported that chronic restraint stress induces depression-like behavior in rodents^14^, and depression is known to promote decreased arterial pulsatility and cerebral blood flow in the brain^15–18^. These results suggest that chronic stress can decrease cerebro-vascular responses causing decreased hemodynamics. In theory, sustained reduction of cerebral hemodynamics can eventually lead to hypoxic conditions, which can affects neuronal deaths and cognitive dysfunction. However, these points have not been clarified yet.

Hypoxic condition results in an upregulation of hypoxia-inducible factor-1α (HIF-1α), which in turn increases the release of vascular endothelial growth factor (VEGF), a primary cytokine involved in angiogenesis. Thus, we hypothesized that chronic stress-induced hypoxic conditions leads to an increase in VEGF and that an increase in VEGF affects vessel structure plasticity, resulting in changes in capillary density^19^. Consistent with the hypothesis stated above, chronic restraint stress has been reported to result in increased HIF-1α expression and higher levels of VEGF and its receptor VEGFR2^20–22^. Furthermore, VEGF levels in the cortex have been found to increase following corticosterone exposure, which mimics stress conditions^23^. However, there is no direct *in vivo* study to show cerebral blood volume changes in conjunction with VEGF and HIF-1α in restraint-rodent model of chronic stress.

In addition to above mentioned VEGF and HIF-1α, claudin-5 and occludin, two major tight junction proteins, have been documented to be reduced in the frontal cortex and hippocampus in a rat model of restraint stress^24^. Alterations in these proteins can be a direct mechanism for blood-brain barrier (BBB) permeability changes. In central nervous system (CNS), BBB is existing to act as selective barrier isolating CNS parenchyma from the circulatory system^25^. BBB integrity is maintained by tight junctions among brain endothelial cells^26^. Also, BBB integrity is related with VEGF. VEGF has been reported to enhance BBB permeability^27–29^. BBB disruption can facilitate the infiltration of pro-inflammatory cytokines and neurotoxic molecules into neural tissue, along with immunoglobulin and albumin, and therefore BBB permeability changes could be a driving force of the vicious cycle of the adverse effects of chronic stress on the brain.

Attempts have been made to evaluate BBB permeability changes under chronic stress^30–33^. However, mixed results have been reported with some studies reporting that prolonged exposure to stress can affect the increases in BBB permeability^34,35^ and others reporting no effect of chronic stress on BBB permeability^33,36,37^. Most studies that have investigated the correlation between stress and BBB permeability have used an endpoint assay, i.e., extravasation of Evans blue or sodium fluorescein shown with tissue slice imaging and colorimetric quantification^31,33,38,39^. These methods may incorrectly overestimate the signal of Evans blue or sodium fluorescein in tissue due to insufficient perfusion power to remove all residual dyes in blood vessels^31^. In addition, the permeability of the BBB is affected by the size, hydrophobicity, and charge of molecules. Therefore, in order to better assess the degree of BBB permeability changes due to chronic stress, it is necessary to perform real-time concomitant measurements of BBB permeability by using different sizes of tracers. Recently, two-photon (2p) imaging has been used to visualize and assess the cerebro-vascular structure and BBB permeability in live animals^40,41^. The implantation of a chronic cranial window enables repeated brain imaging within the same subject and therefore provide clearer picture of long-term effects of chronic stress on the brain system than before^42,43^.

In this study, the long-term effects of chronic stress on the cerebro-vascular volume and BBB permeability are investigated using longitudinal *in vivo* 2p dynamic imaging and we show that chronic restraint stress decreases the size of cerebral vessels and increases BBB permeability, accompanied with up-regulation of VEGF and down-regulation of claudin-5 in mRNA level.

## Results

### Effects of chronic stress on behavior, weight, corticosterone level, and blood pressure

**Figure 1:**
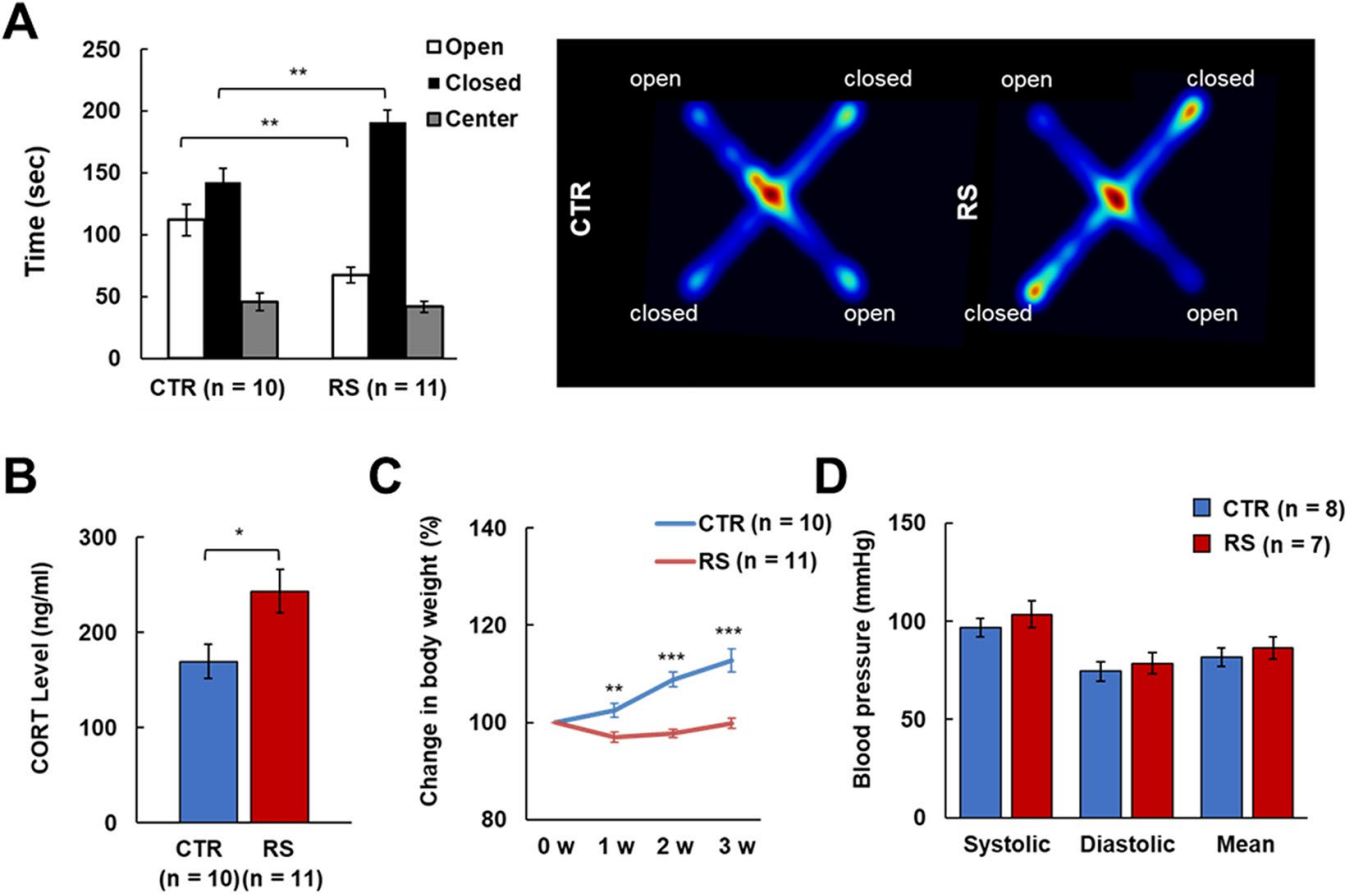
Validation of the chronic restraint stress (RS) animal model. (**A**) Cumulative time in open, closed, and center areas in the elevated plus maze (EPM), and heat map of animals’ movements on the EPM. (**B**) The level of plasma corticosterone (CORT) one day after the last stress exposure. (**C**) Body weight changes over the 3-week period. (**D**) Blood pressure one day after the last stress exposure. CTR, control group; RS, restraint stress group; *p < 0.05; **p < 0.01; ***p < 0.001.

The elevated plus maze (EPM) test confirmed that the 3-week administration of restraint stress (RS) induced behavioral despair (Fig. 1A). The RS animals stayed in the closed area for significantly longer than the control animals, which is a typical depressed-like stress behavior^44,45^. In addition, RS animals showed higher corticosterone plasma levels and a lower weight gain compared to control animals (Fig. 1B,C)^46,47^. Blood pressure (BP) was not significantly different between control and RS animals following the 3-weeks RS stress paradigm (Fig. 1D).

### Effects of chronic stress on cerebro-vasculature

**Figure 2:**
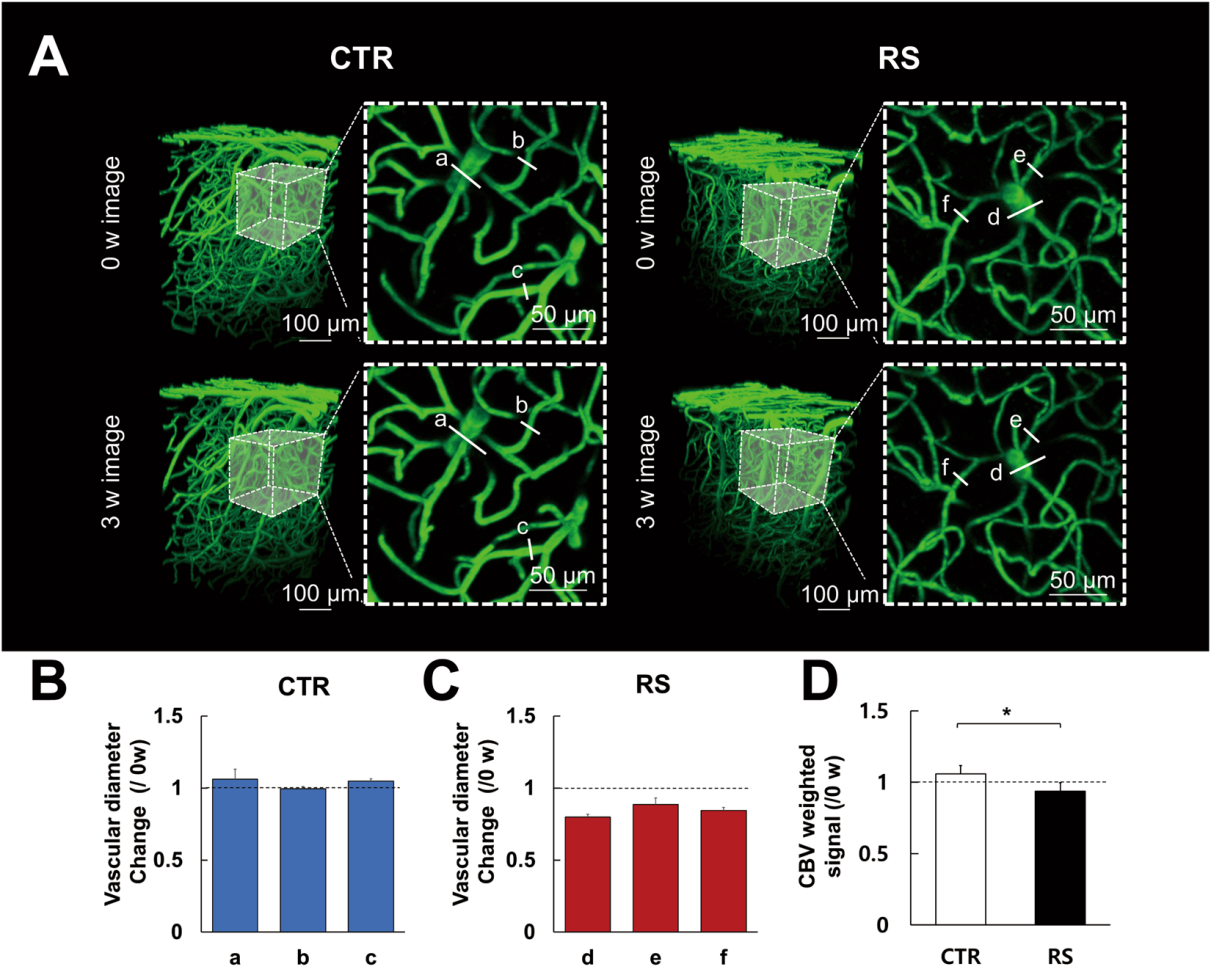
Cerebro-vascular structure following chronic restraint stress. (**A**) Representative images were longitudinally acquired in the control and RS groups. Two-dimensional images (dashed square) are the maximum intensity projection of 3D images with a 100 μm thickness (dashed cube). a-f indicate vessel diameter. Vascular diameter changes of representative images in the CTR (**B**) and RS (**C). **(**D**) Cerebral blood volume (CBV) weighted intensity signal at 3 w shown as the ratio to 0 w. CTR, control group; RS, restraint stress group; *p < 0.05.

Representative images of the cerebro-vasculature taken before and after RS modeling are shown for the control and RS groups (Fig. 2A). The control group exhibited no significant diameter changes in all vessels at 3 weeks (3 w) compared to 0 week (0 w). In contrast, the RS group revealed a decreased diameter of all vessels at 3 w compared to 0 w (Fig. 2A–C, see Supplementary Fig. S1 for raw data).

Since the sum of voxels of the acquired image represents the CBV weighted signal of the region of interest (ROI), the total CBV weighted signal was estimated by measuring the volume of the fluorescent signal of ROI. When we performed within-group comparison of CBV weighted signal between 0 w and 3 w, no statistically significant change was observed in the control group (3w/0w = 105.86 ± 5.78% (mean ± SD); p = 0.077). However, in the RS group, the CBV weighted signal was reduced at 3 w compared to 0 w (3w/0w = 93.65 ± 6.71%, p = 0.049). In addition, the RS group had a significantly lower total CBV weighted signal of vessels compared to the control group at 3 w (p = 0.008, Fig. 2D).

**Figure 3:**
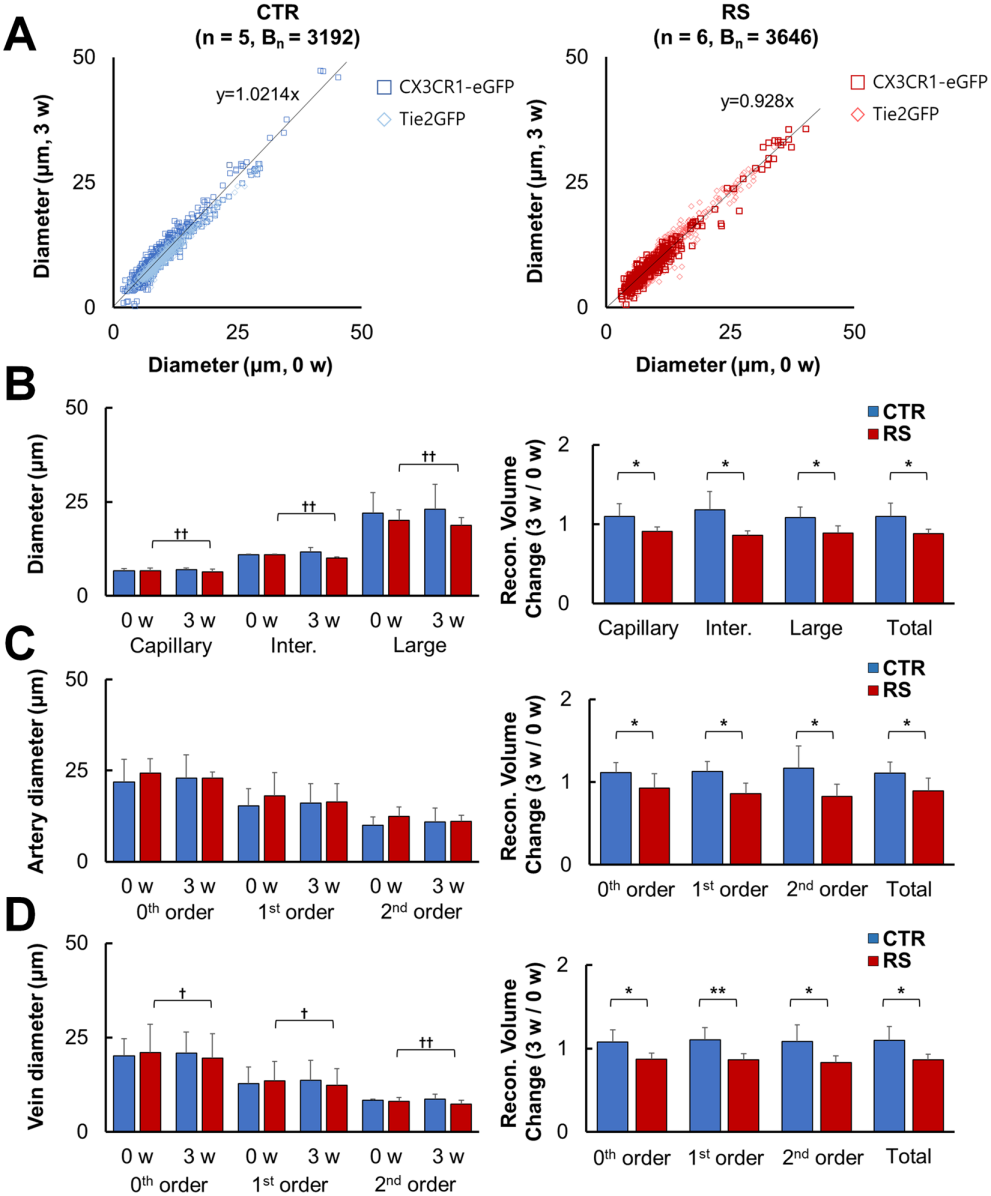
Volume and diameter of classified vessels based on size, type, and branching order. (**A**) The correlation of vessel diameter between 0 w and 3 w of the CTR (left) and RS (right). (**B**) The diameter of vessel at 0 w and 3 w in all sizes of vessels (left), and the reconstructed volume change shown as the ratio to 0 w (right). (**C**) Artery diameter (left) and reconstructed volume change (right) in the CTR and RS based on branching order. (**D**) Vein diameter (left) and reconstructed volume change (right) in the CTR and RS based on branching order. CTR, control group; RS, restraint stress group; B_n_, number of branches; *p < 0.05; **p < 0.01; ^†^p < 0.05; ^††^p < 0.01.

To quantify these structural changes in detail, we performed developed 3D vessel analysis methods mentioned in methods section (see Supplementary Fig. S2 for details). The diameters of each vessel type in the two groups were plotted between 0 w and 3 w (Fig. 3A). The linear regression trend line based on plotted vessels showed no changes between 0 w and 3 w in control group, i.e., the slope is close to one. On the other hand, the RS group showed a significant within-group decrease in slop of trend line between 0 w and 3 w. When we compare mean value of classified segments based on the size of diameter, there was no change in the control group at 3 w vs. 0 w, whereas significant decreases were found in the capillary (≤9 μm), intermediate size (≤14 μm) and large (>14 μm) vessel type of RS group at 3 w vs. 0 w. Also, the same tendency was observed when the diameter was converted into the reconstructed volume based on the analysis described in the Methods (Fig. 3B). This reconstructed volume data reflects CBV weighted intensity of selected vessel segments. This also makes it possible to directly compare the changes in selected vessel segments obtained between 0 w and 3 w. As shown in Fig. 2, 3A and B, the constriction response of vessels was prominent in the RS group regardless of vessel size.

When we compared vessel responses in relation to vessel type and branching order, similar results were obtained (Fig. 3C and D, and Tables 1 and 2). Namely, artery diameter in the control group shows increased trend for the all vessel orders at 3 w compared to 0 w, whereas artery diameter in the RS group shows decreased trend for all vessel orders at 3 w compared to 0 w. Similarly, vein diameter in the control group tend to increase for all vessel orders at 3 w compared to 0 w, whereas vein diameter in the RS group significantly decreased for all vessel orders at 3 w compared to 0 w. Consistent with diameter change, reconstructed volume of all shows statistically significant decrease in the stress group, regardless of vessel type and branching order (Fig. 3C and D).

**Table 1:** Estimated diameter and reconstructed volume of selected blood vessels.

	Number of animals	Number of branches	Diameter										Estimated volume ratio							
				Capillary ( ≤ 9 µm)		p-value	Intermediate ( ≤ 14 µm)		p-value	Large ( ≥ 14 µm)		p-value	Capillary ( ≤ 9 µm)		Intermediate ( ≤ 14 µm)		Large ( ≥ 14 µm)		Total	
				0 w	3 w	Intra.	0 w	3 w	Intra.	0 w	3 w	Intra.	3 w/0 w	p-value	3 w/0 w	p-value	3 w/0 w	p-value	3 w/0 w	p-value
CTR	5	3192	Average	6.688	6.886	0.413	10.95	11.708	0.182	22.004	23.028	0.207	1.0972	0.024	1.1822	0.034	1.0805	0.019	1.0974	0.041
			S.D.	0.589	0.499		0.207	1.093		5.464	6.545		0.16123		0.23096		0.13161		0.16462	
RS	6	3646	Average	6.715	6.407	0.008	10.887	10.067	0.001	20.142	18.745	0.006	0.9085		0.8596		0.8835		0.8831	
			S.D.	0.734	0.759		0.19	0.28		2.778	2.13		0.05313		0.05571		0.09656		0.05266

**Table 2:** Estimated diameter and reconstructed volume of selected blood vessels based on vessel type and branching order.

		Number of vessels	Diameter										Estimated volume ratio							
				0th order			1st order			2nd order			0th order		1st order		2nd order		Total	
				0 w	3 w	Intra p-value	0 w	3 w	Intra p-value	0 w	3 w	Intra p-value	3 w/0 w	p-value	3 w/0 w	p-value	3 w/0 w	p-value	3 w/0 w	p-value
Artery	CTR	5	Average	21.853	22.983	0.063	15.246	16.14	0.116	9.903	10.822	0.315	1.112	0.047	1.125	0.01	1.169	0.046	1.1096	0.043
			S.D.	6.193	6.354		4.799	5.238		2.365	3.839		0.125		0.121		0.267		0.12829	
	RS	5	Average	24.235	22.977	0.357	18.006	16.327	0.121	12.347	11.041	0.107	0.925		0.859		0.829		0.8957	
			S.D.	4.04	1.572		6.464	5.034		2.658	1.666		0.174		0.13		0.146		0.15208	
Vein	CTR	5	Average	20.091	20.909	0.321	12.788	13.662	0.201	8.335	8.713	0.43	1.077	0.014	1.106	0.006	1.085	0.016	1.099	0.028
			S.D.	4.658	5.489		4.338	5.227		0.362	1.262		0.146		0.144		0.196		0.163	
	RS	6	Average	21.067	19.537	0.022	13.467	12.305	0.023	8.164	7.398	0.003	0.87		0.864		0.829		0.862	
			S.D.	7.385	6.48		5.188	4.429		0.971	0.959		0.077		0.074		0.079		0.068	

### Effects of chronic stress on the dynamic BBB permeability

**Figure 4:**
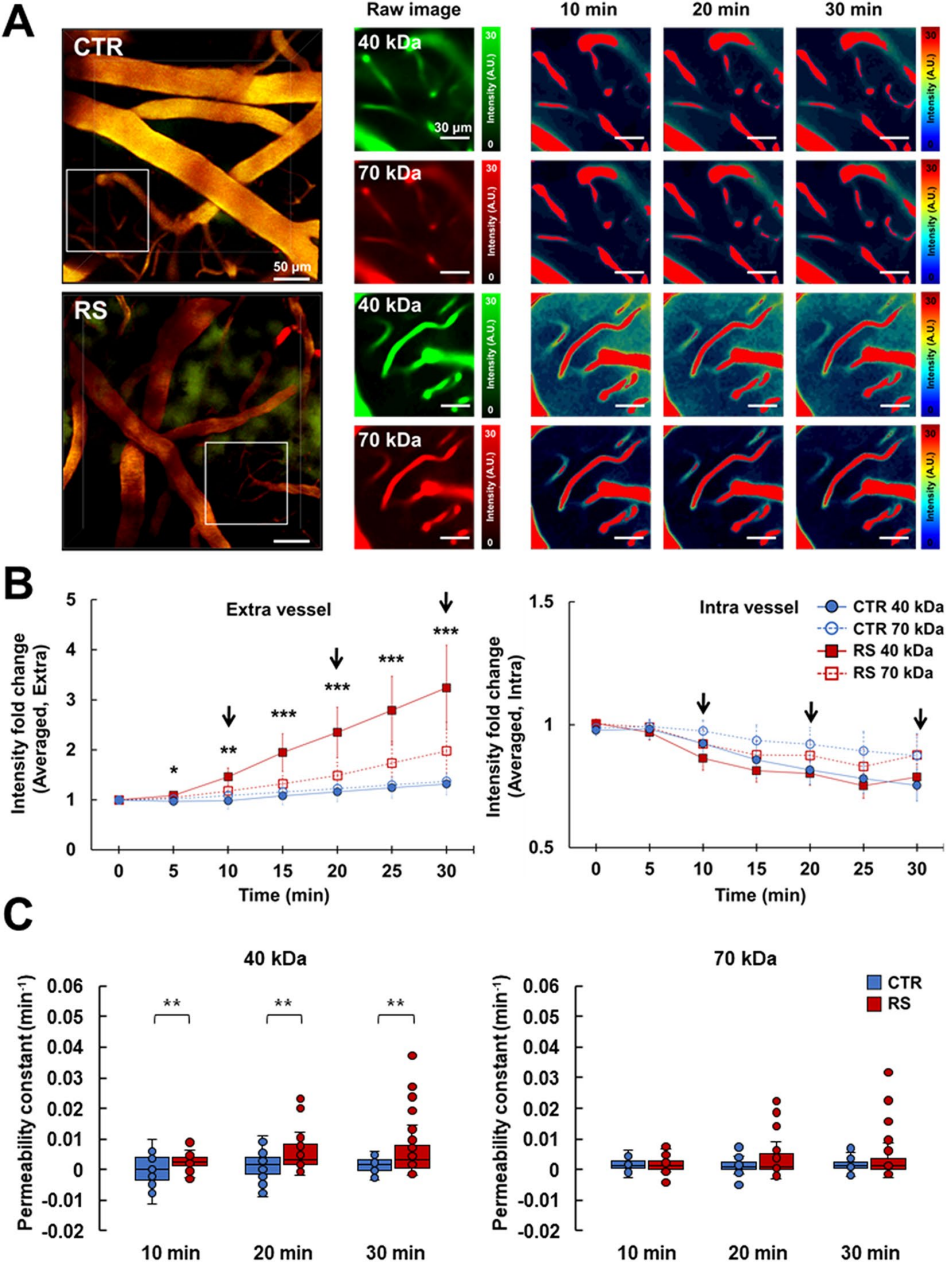
BBB permeability increases following chronic restraint stress. (**A**) Representative images of leaking fluorescence-labeled dextran. The middle panel shows the raw images of the insert squares in the left panel. Time-lapse raw images were visualized with the color intensity scale, as shown in the right panel. (**B**) The intensity of fluorescence in the extravessel (left) and intravessel (right). The intensity of the 40-kDa tracer significantly increased in the extravessel area of the RS from 0 to 30 min. Arrows indicate time points for time-lapse raw images in right panel of A. (**C**) The permeability constant of the 40-kDa tracer (left) and 70-kDa tracer (right). CTR, control group; RS, restraint stress group; *p < 0.05; **p < 0.01; ***p < 0.001.

Two different molecular sizes of fluorescence-labeled dextran were delivered via intravenous injection, and the extravasation of fluorescent signals was measured simultaneously using *in vivo* 2p imaging (Fig. 4A). We monitored the leakage of dextran for 30 min after the injections, and we observed clear leakage of fluorescent dye into the perivascular area, with the extent depending on the size of the molecule. Extravasation of representative images significantly increased in the RS group following the 40-kDa FITC-conjugated dextran injection, but not the 70-kDa Texas-red conjugated dextran injection, compared to the control group (Fig. 4A). As shown in Fig. 4B, 70-kDa dextran showed about 1.5 times increase in RS group and about 1.2 times increase in control group at extravascular intensity after 30 min. Meanwhile, 40-kDa dextran showed about 3 times increase in RS group and about 1.2 times increase in control group at extravascular intensity after 30 min. The intravascular intensity was maintained at the similar level between RS and control groups in 40-kDa and 70-kDa dextran injection for 30 min.

The permeability constant calculated by our modified formula revealed an increased permeability of the BBB to the 40-kDa dextran but not to the 70-kDa dextran in the RS group compared to the control group (Fig. 4C, full time video in Supplementary Video S1).

### Expression of mRNA related to the BBB

**Figure 5:**
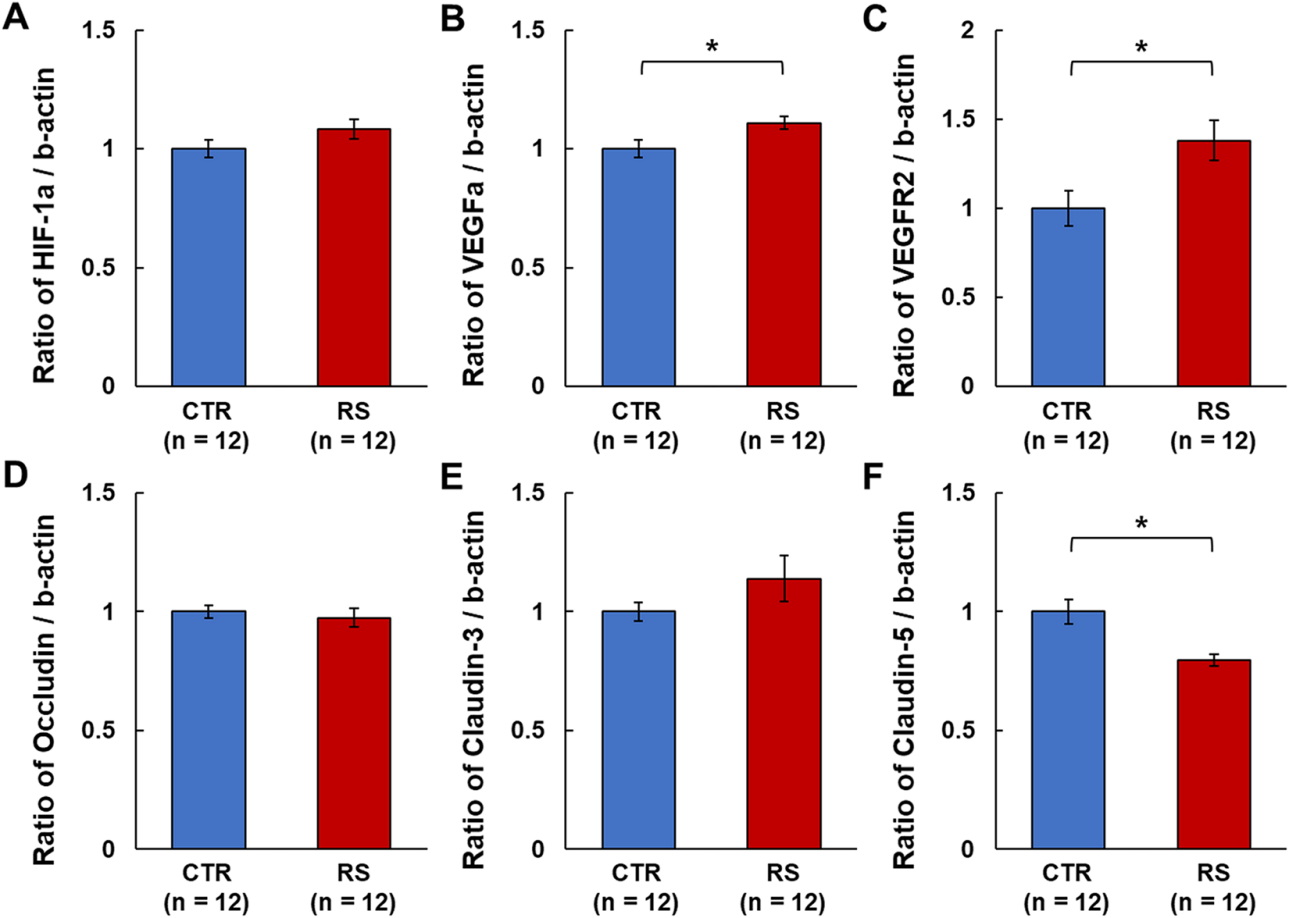
mRNA expression of hypoxia related factors and tight junction proteins underlying BBB permeability. (**A**–**C**) The expression of hypoxia related factors, HIF-1α, VEGFa, and VEGFR2 in the somatosensory cortex. (**D–F**) The expression of tight junction proteins, occludin, claudin-3, and claudin-5 in the somatosensory cortex. CTR, control group; RS, restraint stress group; *p < 0.05.

Prolonged reduction of cerebral blood volume, as observed via the lower vessel intensities in the RS group, can lead to a lack of oxygen for brain parenchyma. This hypoxic condition can induce alterations in HIF-1α and VEGFa expression. There was an increase in HIF-1α mRNA expression that nearly reached significance in the RS group (*P = *0.0506), and the HIF-1α target gene, VEGFa, and VEGFR2 mRNA had significantly higher expression levels in the RS group compared to the control group (Fig. 5A–C). The mRNA expression of occludin and claudin-3 did not differ between the RS and control groups (Fig. 5D,E). In contrast, claudin-5 was significantly lower in the RS group compared to the control group (Fig. 5F).

## Discussion

In the present study, we investigated whether repeated restraint stress induced decreases in cerebro vascular volume and BBB permeability in mouse cortex with *in vivo* 2p imaging. Even though there have been a few studies for cerebro-vascular alteration of RS animal, there is no study providing direct comparison of cerebro-vasculatures before and after the stress regime. Also, whether chronic stress affects BBB permeability is still questionable, particularly for intermediate-sized molecules^33–37^.

By utilizing real-time *in vivo* 2p imaging, we found that chronic RS induced the decreases in the diameter of all vessel type, reconstructed volume of the selected cerebral vasculature and increased BBB permeability for intermediate-sized molecules. In addition, our chronic RS mice showed behavioral changes and increased plasma corticosterone level, which was typical findings in stressed animals consistent with previous study^14,46,47^. It was reported that corticosterone is related with the regulation of heme oxygenase-2 (HO-2) and nitric oxide synthase (NOS) in the rat brain^48^. Chronic stress induces the corticosterone induction followed by the reduction of NOS and HO-2 expression^12^ and eventually the reduction of nitric oxide (NO)^49–51^ and carbon monoxide (CO), major vasodilators in the brain. Thus, these chain reactions would inevitably result in the reduction of cerebro-vascular volume and affects overall hemodynamics in the cortex.

Vascular volume reduction caused by RS was confirmed in this study from 2p fluorescent imaging data. Indeed, vascular volume reduction, based on volume-reconstruction of selected vessel segment, was found for all types of blood vessels, i.e., artery and vein, regardless of vessel size and branching order in stressed animals compared to control animals. However, since the animals were under anesthesia, there was a chance that anesthesia might affect the outcome of this study. Indeed, the effect of anesthesia on neurovascular regulation has been extensively investigated^52^ and even isoflurane has been reported to dilate blood vessels^53,54^.

To minimize the influence anesthesia level fluctuation on vascular dynamics, we ensured stable anesthetic condition by frequent monitoring of the pedal reflex and respiratory pattern during imaging. In addition, we also tracked anesthesia level during imaging by monitoring heart rate (HR). As shown in the Supplementary Fig. S5, no significant change in HR was observed during imaging. Still, there was a potential for a certain level of fluctuation in anesthesia depth caused by different dosage and different kind of anesthesia agents, which might have affected the measurements. Therefore, we explored whether the depth of anesthesia (e.g., % isoflurane), different kind of anesthesia actually does affect the vessel diameter measurements.

First, images were repeatedly acquired with the anesthetic concentration of 1.5% isoflurane for either 30 minute or 60 minute. During these prolonged anesthesia, the change of vessel diameter was checked in naïve animals and stressed animals respectively to ensure whether vaso-fluctuations is prominent. Our result confirmed that there were no significant fluctuation in vessel diameter for both naïve condition and stressed condition. (Supplementary Fig. S3, S4). In addition, when anesthetic concentration was increased to 2% isoflurane in stressed animals’ imaging, there was also no significant fluctuation in vessel diameter (Supplementary Fig. S3, S4). These results indicate the prolonged anesthesia at 1.5% or 2% isoflurane will not cause significant fluctuations in vessel diameter. Although there was some vaso-dilation due to changes in anesthesia level (1.5% to 2%), a larger pattern emerges when comparing pre-stress images to those after chronic stress regime (Supplementary Fig. S3, S4). Additionally, we have confirmed that alternative ketamine-xylazine anesthesia results in consistent results with results from isoflurane anesthetic condition (Supplementary Fig. S6), supporting that the chronic stress shrinks blood vessels.

There are several studies showed decreased cerebral blood volume (CBV) following chronic stress in animals and humans^1,11–13^. Prior exposure to chronic stress was reported to occlude strong activation of the somatosensory cortex by CO_2_ inhalation using functional Magnetic Resonance Imaging (fMRI) and to exhibit a decreased hemodynamic response in the somatosensory cortex during hind paw electrical stimulation using optical intrinsic signal imaging^11,12^. According to the Poiseuille’s law of fluid dynamics, i.e., flow proportional to the fourth power of vessel radius, assuming that the blood pressure within the vessel is maintained, this would lead to ~23% reduction in cerebral blood flow, which could significantly impact the neurovascular regulation and brain metabolism^55^. Consistent with mRNA analysis, the velocity of RBC was significantly decreased in capillary vessels of chronically stressed animals compared to the naïve animal prior to the stress regime. (Supplementary Fig. S8). More research is needed, but we suspect that the cause of RBC velocity decline is due to increased resistance of the vessel wall and decreased blood flow due to contraction of blood vessel. Inadequate CBF and CBV can result in insufficient oxygen supply; considering that neurons and glial cells receive oxygen from blood vessels, a reduced CBV may induce hypoxic condition and trigger HIF-1a expression and sequential expression of VEGF^56,57^. These two molecules are typical molecules in the hypoxic state and are known to affect angiogenesis in particular^57^. Under cerebral ischemia, VEGF enhanced angiogenesis when administration at late stage but increased BBB leakage at early stage^27^. In inflammatory CNS condition such as multiple sclerosis (MS) stress condition, astrocytic expression of VEGF-A is reported to a key driver of BBB permeability in mice^58^. Stress also induces neuroinflammation^59–61^. Under inflammatory condition, VEGF-A induces BBB disruption than angiogenesis.

Our results showed significantly higher HIF-1α, VEGFa, and VEGFR2 mRNA expression level in chronically stressed brain than control brain. VEGF and its receptors, particularly VEGFR2, are essential regulators of patterning the vessel structures in development and vascular permeability^62,63^. Extensive research has focused on the relationship between VEGF and stress. These studies reported diverse changes in VEGF expression levels in relation to stress type and brain area^22,23,64,65^. For example, oral administration of corticosterone for 7 weeks or treatment of cultured neuronal cells increased VEGF protein levels and decreased Flk-1 protein (VEGFR2) levels^23^. Researchers have shown that acute restraint stress increased VEGF protein and mRNA levels in the ascending aorta and endothelial cell proliferation, which was modulated by nerve growth factor and its receptor^66^. Based on our results and the results of these papers, it can be inferred that chronic stress causes a decrease in cerebro-vascular volume and thus increases in levels of HIF-1α and VEGF. In addition, while reduced cerebro-vascular volume could increase systemic blood pressure as a compensation mechanism, it is controversial whether stress affects systemic blood pressure^5,67,68^ and we found no changes in systemic blood pressure following chronic RS.

In addition, our results showed downregulation of mRNA levels of claudin-5, one of the tight junction proteins, but not that of occludin or claudin-3. Previous studies have reported that claudin-5 is more sensitive to stress conditions than occludin^24^ and that chronic stress significantly reduced the level of claudin-5 but not that of occludin^32^. Over-expression of VEGFa and down-expression of claudin-5 are closely related to BBB permeability^29^.

Therefore, we evaluated the BBB permeability in chronically stressed brains using two different sizes of fluorescence-labeled dextran (40-kDa as intermediate size and 70-kDa as large size) and real-time *in vivo* 2p imaging. For the BBB permeability study, we used thinned skull window, which is advantageous for minimally invasive imaging of the cortical surface; however, regrowth of the skull limits the number of observation and imaging depth in longitudinal study^69^. As BBB permeability can be measured at the cortical surface and does not require repeated measurements, we chose thinned-skull window for the permeability study. The extravasation of 40-kDa FITC dextran was significantly increased in the chronic RS group compared to the control group, but no significant changes were observed for 70-kDa dextran. This confirmed results from a previous study using a 70-kDa size tracer^33^.

Several papers have reported that stress induces higher BBB permeability in mice^70,71^ but others have suggested otherwise^31,39^. This difference may have been caused by using different kinds of tracers. Two papers showed stress-induced BBB permeability changes with pyridostigmine (181-Da)^70^ and 99mTc (98-Da)^71^ as tracers, and two other papers found no relation of stress to BBB permeability with Evans blue (68 kDa)^31,39^. A recent publication reported that chronic stress does not increase BBB permeability in mice to sodium fluorescein (376-Da) and FITC dextran (70-kDa)^33^. Generally, the BBB occludes molecules over 400-Da. These results appear to be dependent on the tracer, particularly the size.

Body temperature has also been reported to be an important factor in the regulation of BBB permeability^33^. In our study, the animal’s body temperature was kept at 37 °C using a heating pad during the live 2p imaging. Because most of previous studies are tissue-based studies postmortem, this study may provide more information about BBB permeability under real live conditions. As 2p imaging can assess dynamic changes over a long period of time, this study could provide valuable information in relation to not only the time-course of molecular leakage but also better assessment of BBB permeability changes in brain tissue. To summarize our BBB results, chronic RS increased BBB permeability but did not induce BBB disruption, which was assessed using the 70-kDa tracer. These increases in BBB permeability could be due to the decreased expression of the tight junction protein, claudin-5, possibly resulting from increased expression of VEGFa and VEGFR2.

Our study are limited on only cerebral cortex because of using 2p imaging with live animals. Many studies have reported the effect of stress on hippocampus but hippocampus is hard to study using 2p imaging with live animals owing to the current technical limitation. Stress animal models are diverse in strain of animal, stressor, and duration. We studied only one kind of stress animal model and we displayed only small information about stress. Previously, many researchers also have studied different kind of stress animal models and a few studies focused BBB permeability. In rat model of stress, it was reported that chronic unpredictable stress for 10 days and acute immobilization stress for 20 minutes had no change in BBB permeability^32,38^. Early life stress, such as prenatal stress (E10–20) and postnatal stress (P2–20), was reported to induce BBB disruption in rat brain by increasing caveolae-mediated transport in brain endothelial cells^34^. In mice model of stress, acute forced swim stress was reported to induce no significant changes in extravasation of sodium fluorescein (376-Da) and FITC-dextran (70-kDa)^33^. On the other experiment with mice model, it was reported that acute immobilization stress for 30 minutes induced BBB disruption in hippocampus, diencephalon, cerebellum, and brainstem demonstrating involvement of CRH (corticotropin releasing hormone) and mast cells increases in regulating BBB permeability^71^. Therefore, more studies using more variety of stress models should be made in the future. A better understanding of stress to the cerebro-vascular changes based on diverse stress model and methodology will aid the development of novel methods to restore vascular plasticity in stress-related neurodegenerative and neurological diseases.

In sum, this study provides empirical evidence of alterations in cerebro-vascular volume and BBB permeability induced by chronic stress. Namely, prolonged exposure to RS may lead to a decrease in the diameter of all types of blood vessels and a decrease in reconstructed volume of selected vessel, and that these changes could be a driving force for re-shaping the neurovascular structure as well as BBB permeability.

## Methods

### Animal Care

All experimental processes conformed to the national guidelines of the Korean Ministry of Food and Drug Safety on the care and use of laboratory animals and were approved by the Institutional Animal Care and the Use Committee (IACUC, Permit #: SKKUIACUC 2016-05-0002-2) of the Sungkyunkwan University. The animals were caged in an environment maintained with a 12 hour inverted light/dark cycle (lights on at 9:00 pm), at a temperature of 24–25 °C, and 50–60 % humidity. Male mice 9–11 weeks old were used for this study. Tie2GFP (control: n = 2; RS: n = 4; STOCK Tg(TIE2GFP)287Sato/J (Stock No: 003658, Jackson Laboratory) and CX3CR1-eGFP (control: n = 3; RS: n = 2; B6.129P-*Cx3cr1*^*tm1Litt*^/J (Stock No: 005582, Jackson Laboratory) mice were used for repeated imaging of the cerebral vasculature. After cranial window surgery, mice were reared in individual cages with a recovery period of 4–6 weeks before imaging. Wild-type C57BL/6 mice (OrientBio, South Korea) were used for BBB permeability imaging (control: n = 3; RS: n = 3) and blood pressure measurements (control: n = 8; RS: n = 7).

### Restraint stress model

To induce chronic restraint stress, mice were immobilized with a plastic bag (Decapicones, Braintree Scientific Inc.) in their home cages 6 hours per day for 3 weeks. The time of stress administration was fixed in the morning (9:30 am). During stress exposure, food and water were restricted. Mice in the control group were not restrained and remained in their home cages during this 3-week period. Body weight and food intake were monitored weekly. In the RS group, all imaging experiments and brain tissue and blood sample collection were performed one day after the last stress exposure.

### Chronic cranial window surgery for *in vivo* 2p microscopic imaging

To perform *in vivo* 2p microscopic imaging of the brain, animals underwent a cranial window installation surgery. Before the installation, animals were anesthetized by isoflurane inhalation (MIP Company, OR). Body temperature was maintained at 37 °C by a homeothermic heating pad system (FHC, ME), which was controlled by a rectal probe. The isoflurane level was 3 % for the initial anesthesia induction and maintained at 1.5 % during the cranial window surgical procedure. Heart rate and SpO_2_ of animals were monitored throughout the entire procedure to ensure physiological health (PhysioSuite, Kent Scientific, CT). During the window installation procedure, animals were fixed in a stereotaxic frame (David Kopf Instruments, CA). A cranial window 3 mm in diameter was made in the right hemisphere and centered at ML, +2.5 mm, AP, −1.5 mm. A customized chamber frame (Narishige, Tokyo, Japan) was placed around the opened skull and fixed with cyanoacrylic glue. The exposed cortex was covered with a 4-mm glass coverslip (Warner instruments, CT), which was fixed with cyanoacrylic glue. The rest of the cranial window margin and skull area were filled with dental resin. After the window installation surgery, the animals were injected with enrofloxacin (Baytril, anti-biotic) and meloxicam (Metacam, anti-inflammatory and analgesic drug) and underwent a 4–6 week recovery period before imaging experiments to avoid any confounding neuro-inflammatory effects on imaging data. We found that cranial windows could be maintained for 4–5 months.

### Longitudinal vasculature imaging using *in vivo* 2p microscopy

The RS group was imaged using 2p microscopy (TCS SP8 MP, Leica Microsystems, Germany) before stress exposure and at the end of the 3-week stress exposure. The control group was also imaged twice, with a 3-week interval. For *in vivo* 2p imaging, mice were anesthetized with 3 % isoflurane for induction and 1.5 % isoflurane for maintaining anesthesia state during imaging. After confirmation of proper anesthesia, mice were placed on a head-fixing apparatus (MAG-1, Narishige, Japan) under a 2p microscopic imaging system. Body temperature was maintained at 37 °C by a homeothermic heating pad system (FHC), which was controlled by a rectal probe. Fluorescein isothiocyanate (FITC)-conjugated dextran 70-kDa (Sigma Aldrich) or Texas red-conjugated dextran 70-kDa (Molecular probes) was delivered via the retro-orbital sinus (5 % dextran solution (1.5 μl/g of body weight)) to image the vessel structure. The brain was excited with an 800 nm or 910 nm Ti:Sapphire tunable femtosecond laser (Chameleon Vison II, Coherent, Inc., Santa Clara, California), and the emitted fluorescent signal was detected by HyD (hybrid detector), which is newly developed from Leica for taking advantages of both photomultiplier tube and the avalanche photodiode, through a 525/40 bandpass filter cube for FITC-conjugated dextran imaging, and a 617/73 bandpass filter cube for Texas-red conjugated dextran. The imaged brain size was 354.29 × 354.29 μm^2^ (512 × 512 or 1024 × 1024 pixels), which was acquired by a 25X/0.95 NA water-immersion objective lens (HCX IRAPO) from Leica. The imaging depth was approximately 400–500 μm from the brain surface with a z-axis resolution of 1 μm.

### Vascular analysis

We used IMARIS 8.2 software (Bitplane, Switzerland) and Fiji (ImageJ) to preprocess longitudinal images. For preprocessing, the acquired images at 0 weeks (0 w) and 3 weeks (3 w) were smoothed by a 3D Gaussian filter with a 1.38 μm full width at half maximum (FWHM) kernel size for background blurring and homogenization of vessel. Then, to enhance the signal-to-noise ratio, background subtraction using a rolling ball algorithm, which is a Fiji plugin, was conducted. After smoothing and background subtraction, z-slice normalization was performed to correct attenuation of scans from the physical depth. This z-slice normalization is performed to adjust the mean and standard deviation (SD) values of individual z-slice images to the grand mean and SD values of the whole image. Next, we corrected non-homogenous illumination of the x-y plane, resulting from a shadow of an apical pial artery and vein, by dividing the preprocessed image into a maximum intensity projection (MIP) image. Specifically, the MIP image was generated by excluding 100 μm of apical surface and then smoothed by a 3D Gaussian filter with a 13.8 μm FWHM kernel size to obtain an intensity nonuniformity (bias) field. Then, we applied the Contrast Limited Adaptive Histogram Equalization (CLAHE) algorithm in the Fiji plugin to enhance micro-vessel contrast^72^. We generated a binary image using a Mexican Hat filter-based thresholding technique, termed “local contrast”, in the IMARIS software (Supplementary Fig. S2A).

We also applied skeletonize and diameter mapping algorithms to obtain information on vascular morphology. First, we calculated the vessel diameter using Euclidean distance transformation of the fitted sphere located at the center point of the binary vessel images^73^. Then, for longitudinal analysis in the same coordinate, each of the 3 w diameter maps were registered to each of the 0 w diameter maps using non-rigid 3D transformation (Supplementary Fig. S2B)^74^. Second, we generated a skeleton of the 0 w binary image using the 3D skeletonize plugin in Fiji and converted it into a network graph based on a 26-cell cubic neighborhood (Supplementary Fig. S2C)^75^. Next, we merged the diameter map with the vessel network map to classify vessel segments into large vessels (>14 μm), medium vessels (≤14 μm), or capillaries (≤9 μm) (Supplementary Fig. S2E-vascular classification). To calculate a volume of the classified vessels, the volume of the all segments was reconstructed using the diameter information from each voxel in each segment under the assumption of that the vessels are in cylinder form. This means that the cylinders, which has a height of voxel resolution, were constructed at the voxels on all network graphs (Supplementary Fig. S2D-volume estimation). Thus, the sum of the cylinder volumes per voxels represents the approximate volume of a vessel segment. The formula used is as follows:

$$SV=\sum _{n=1}^{N}{r}^{2}(n)\pi l$$

$$Reconstructed\,vascular\,volume=\sum _{t=1}^{T}SV(t)$$

where, SV denotes the volume of segments corresponding to the *t-*th segment, and *T* denotes the total number of segments. Subsequently, *r*(*n*) is half the diameter mapped to the *n*-th voxel of the network graph, and *N* is the number of voxels in a segment. *π* is the circumference, and *l* is the resolution of the voxel, which is 1 μm in this analysis. We estimated the approximated regional CBV by using this reconstructed CBV.

Next, to classify vessel type and branching order, the arteries and veins were separated according to the visual observation of the RBC shadow and the micro vascular fluctuation at apical cortex. We manually identified the starting point of the pial artery and the vein network. The diameter and volume of the artery and vein were further quantified by dividing vessels into the 0^th^, 1^st^, and 2^nd^ order branches. The 0^th^, 1^st^, and 2^nd^ order branches were defined by the thickest branch that stemmed from the starting point, the first branch extending from the 0^th^ order, and the next branches, respectively (Supplementary Fig. S2E). The reconstructed volume of selected vessel was calculated in the same way as above for the divided branches, and the change in volume by vessel type was compared in the CTR and RS groups.

### BBB permeability measurement

To measure BBB permeability in the control and RS groups, time-lapse imaging of the pial arteries was acquired followed by an intravenous injection of two molecule size of fluorophore-tagged dextran (40-kDa and 70-kDa). We made an observation window above the somatosensory cortex by thinning the skull to ~30 μm. All procedures were performed very carefully to prevent damage to the cortex and vessel structures. First, animals received 70-kDa Texas-red dextran to visualize vessel structures, and the 2D imaging plane was positioned at the center of the pial vessels under a 2p microscope. Then, 40-kDa FITC dextran was delivered through an intravenous catheter after a 30 sec image acquisition excited with an 820 nm laser. The 512 × 512 images were taken at 1-sec intervals for 30 min with a 25X objective lens. To check the thickness of the thinned skull, reflectance imaging was performed using confocal microscopy with a combined excitation of 488, 568, and 647 nm lasers. The reflected signals were collected through PMT detectors with wavelength ranges of 488 ± 10, 568 ± 10, and 647 ± 10 nm, respectively. To quantify BBB permeability, we applied the modified formula developed by Dreher *et al*. and Nhan *et al*.^76,77^.

$$P({\rm{t}})=\frac{\frac{d{I}_{e}}{{d}_{t}}}{\frac{{I}_{i}(t)}{1-HCT}-\frac{{I}_{e}(t)}{{V}_{e}/{V}_{i}}}$$

where *V*_*e*_ is the volume of the extravascular region, *V*_*i*_ is the volume of the intravascular region, and *I*_*e*_ and *I*_*i*_ are the extravascular and intravascular fluorescence intensities, respectively. HCT is 0.45 and represents the average hematocrit level of all blood vessels within the imaging field of view. *V*_*e*_/*V*_*i*_ is the volume fraction.

To define *V*_*i*_ and *V*_*e*_, we generated intravascular and extravascular mask images using the 70-kDa image. Then, using the mask image, we divided the fluorescence intensity of 70-kDa and 40-kDa dextran into extra- and intravascular intensity. Then, we calculated BBB permeability using the above formula. In general, BBB permeability is known to be high near capillaries and veins^78,79^. Thus, we only considered fluorescence dynamics and BBB permeability at five circular capillary-containing ROIs (25 μm in diameter) per animal.

### Behavioral test

The elevated plus maze (EPM) test was performed one day after the last stress exposure and at equivalent time point in the control group to analyze anxiety-like behaviors of the mice. The plus maze consisted of four arms (32.5 cm × 5 cm). Two arms faced one another. Two opposite arms were enclosed by 20-cm walls (closed platform) and the other two arms were not enclosed by walls (open platform). Movements on the platforms were recorded for 5 min using a video recording and analysis system (Ethovision XT, Noldus, Wageningen, Netherlands). The total time spent in the open platform, closed platform, and center area was automatically calculated using the behavior analysis software.

### Blood pressure

Blood pressure (BP) was measured one day after the last stress exposure, 60 times over 1 hour, using a physiological monitoring system (CODA monitor, Kent Scientific, CT) by attaching a cuff to the tail of the mouse. To prevent the distortion of BP due to mouse movement, the mice were anesthetized with ketamine and xylazine (100 mg/kg and 10 mg/kg, IP) 5 min prior to BP measurements.

### Measurement of blood plasma corticosterone levels

After the 3-week RS paradigm, mice were briefly anesthetized with 3 % isoflurane, and approximately 200 µl of blood was collected in heparin-coated tubes (BD Vacutainer, Becton Dickinson, NJ). The blood samples were centrifuged at 13,000 rpm for 15 min at 4 °C. The concentration of corticosterone in plasma was analyzed using a corticosterone ELISA kit (Assaypro LLC, MO). The absorbance at 450 nm was measured using a microplate reader (Synergy HT Multi-Mode Microplate Reader, BioTek Instruments, Inc., VT). A standard curve was generated using standard solutions, and the plasma corticosterone level was determined from the standard curve.

### Quantitative real-time PCR

Total RNA was isolated from the somatosensory cortex of a coronal section using an RNeasy Mini Kit (Qiagen, Hilden, Germany), and the concentration of RNA was measured using a Take3 Micro-Volume plate/Synergy HT Multi-Mode Microplate Reader (BioTek Instruments, Inc., VT). cDNA synthesis was completed using a High Capacity RNA-to-cDNA Kit (ThermoFisher, MA). Quantitative real-time PCR was performed in duplicate with specific primers (Supplementary Table S2) using SYBR Green PCR Master Mix (ThermoFisher, MA) and QuantStudio 3 Real-Time PCR System (ThermoFisher, MA). The real-time PCR cycle consisted of 1 cycle at 95 °C for 10 min, followed by 40 cycles at 95 °C for 15 sec and 60 °C for 1 min. A melting curve analysis was conducted at the end of the real-time PCR reaction for each specific primer pair. The values were calculated as relative changes to the control after normalization to the beta-actin gene.

### Statistics

We validated the normal distribution of all data through the Shapiro-Wilk test and then divided it into Independent Student’s t-test and Mann-Whitney U test according to the results of normality test. Independent Student’s *t*-test was performed to ascertain statistical significance in behavior test, the level of plasma corticosterone, body weight changes, blood pressure, most of vascular diameter and volume, intensity fold change in BBB permeability and mRNA expression between control and RS group. Mann-Whitney U test was used to confirm the statistical significance of permeability constant and 0^th^ order artery between two groups (control and RS). We also used paired Student’s *t*-test to confirm the statistical significance of changes in the vascular diameter and volume within groups. Data are expressed as the mean ± SD except mRNA expression as the mean ± SE. Statistical significance was set at p < 0.05. Statistical analysis was performed using the SPSS (IBM SPSS statistics 20, NY).

## Electronic supplementary material

Supplementary Information

Representative in vivo real time imaging of the extravasation of fluorescence-labeled dextran (40-kDa and 70-kDa)

## Data Availability

All data are available from the corresponding authors upon request.
