# Supplementary material for: Real-time in vivo two-photon imaging study reveals decreased cerebro-vascular volume and increased blood-brain barrier permeability in chronically stressed mice
Source: Sci Rep. 2018 Aug 30;8:13064. doi: 10.1038/s41598-018-30875-y (PMC6117335; doi:10.1038/s41598-018-30875-y)
Supplement: Supplementary file 1 — Supplementary Information [file 41598_2018_30875_MOESM1_ESM.pdf]

Real-time *in vivo* two-photon imaging study reveals decreased cerebro-vascular volume and increased blood-brain barrier permeability in chronically stressed mice

Sohee Lee [1†], Bok-Man Kang[1,2†], Jae Hwan Kim[1,3†], Jiwoong Min[1,2], Hyung Seok Kim[1,2], Hyunwoo Ryu[1,2], Hyejin Park[1,2,5], Sungjune Bae[1,2], Daehwan Oh[3], Myunghwan Choi[1,2,3] & Minah Suh[1,2,3,4*]

Supplementary methods, figures, video and table

Supplementary methods

Chronic cranial window surgery for in vivo 2p microscopic imaging

To perform *in vivo* 2p microscopic imaging of the brain, animals underwent a cranial window installation surgery. Before the installation, animals were anesthetized by isoflurane inhalation (MIP Company, OR). Body temperature was maintained at 37 °C by a homeothermic heating pad system (FHC, ME), which was controlled by a rectal probe. The isoflurane level was 3 % for the initial anesthesia induction and maintained at 1.5 % during the cranial window surgical procedure. Heart rate and SpO_2 of animals were monitored throughout the entire procedure to ensure physiological health (PhysioSuite, Kent Scientific, CT). During the window installation procedure, animals were fixed in a stereotaxic frame (David Kopf Instruments, CA). A cranial window 3 mm in diameter was made in the right hemisphere and centered at ML, +2.5 mm, AP, -1.5 mm. A customized chamber frame (Narishige, Tokyo, Japan) was placed around the opened skull and fixed with cyanoacrylic glue. The exposed cortex was covered with a 4-mm glass coverslip (Warner instruments, CT), which was fixed with cyanoacrylic glue. The rest of the cranial window margin and skull area were filled with dental resin. After the window installation surgery, the animals were injected with enrofloxacin (Baytril, anti-biotic) and meloxicam (Metacam, anti-inflammatory and analgesic drug) and underwent a 4 - 6 week recovery period before imaging experiments to avoid any confounding neuro-inflammatory effects on imaging data. We found that cranial windows could be maintained for 4 - 5 months.

Behavioral test

The elevated plus maze (EPM) test was performed one day after the last stress exposure and at equivalent time point in the control group to analyze anxiety-like behaviors of the mice. The plus maze consisted of four arms (32.5 cm x 5 cm). Two arms faced one another. Two opposite arms were enclosed by 20-cm walls (closed platform) and the other two arms were not enclosed by walls (open platform). Movements on the platforms were recorded for 5 min using a video recording and analysis system (Ethovision XT, Noldus, Wageningen, Netherlands). The total time spent in the open platform, closed platform, and center area was automatically calculated using the behavior analysis software.

Blood pressure

Blood pressure (BP) was measured one day after the last stress exposure, 60 times over 1 hour, using a physiological monitoring system (CODA monitor, Kent Scientific, CT) by attaching a cuff to the tail of the mouse. To prevent the distortion of BP due to mouse movement, the mice were anesthetized with ketamine and xylazine (100 mg/kg and 10 mg/kg, IP) 5 min prior to BP measurements.

Measurement of blood plasma corticosterone levels

After the 3-week RS paradigm, mice were briefly anesthetized with 3 % isoflurane, and approximately 200 µl of blood was collected in heparin-coated tubes (BD Vacutainer, Becton Dickinson, NJ). The blood samples were centrifuged at 13,000 rpm for 15 min at 4 ℃. The concentration of corticosterone in plasma was analyzed using a corticosterone ELISA kit (Assaypro LLC, MO). The absorbance at 450 nm was measured using a microplate reader (Synergy HT Multi-Mode Microplate Reader, BioTek Instruments, Inc., VT). A standard curve was generated using standard solutions, and the plasma corticosterone level was determined from the standard curve.

Quantitative real-time PCR

Total RNA was isolated from the somatosensory cortex of a coronal section using an RNeasy Mini Kit (Qiagen, Hilden, Germany), and the concentration of RNA was measured using a Take3 Micro-Volume plate / Synergy HT Multi-Mode Microplate Reader (BioTek Instruments, Inc., VT). cDNA synthesis was completed

using a High Capacity RNA-to-cDNA Kit (ThermoFisher, MA). Quantitative real-time PCR was performed in duplicate with specific primers (Supplementary Table S2) using SYBR Green PCR Master Mix (ThermoFisher, MA) and QuantStudio 3 Real-Time PCR System (ThermoFisher, MA). The real-time PCR cycle consisted of 1 cycle at 95 ℃ for 10 min, followed by 40 cycles at 95 ℃ for 15 sec and 60 ℃ for 1 min. A melting curve analysis was conducted at the end of the real-time PCR reaction for each specific primer pair. The values were calculated as relative changes to the control after normalization to the beta-actin gene.

Anesthesia effect test

To clarity the effects of anesthesia on vascular dynamics, we explored whether the depth of anesthesia (e.g., % isoflurane), different kind of anesthesia actually does affect the vessel diameter measurements of *in vivo* 2p microscopic imaging. First, before the stress regime (0 week), the animals underwent anesthesia using the maintenance isoflurane concentration of 1.5 %, and the change of vessel diameter was observed over a 60-minute period to measure vaso-fluctuations during constant-state anesthesia maintenance (Supplementary Fig. S3A&B, S4A&B). The animals were then subjected to the stress protocol. Two weeks later, the stressed animals underwent a 30-minute imaging session, also performed under 1.5 % isoflurane concentration (Supplementary Fig. S3C, S4C). At 30 minutes the isoflurane level was raised to a 2 % concentration (Supplementary Fig. S3C, S4C) to determine how anesthetic condition affects vasoconstriction in stressed model. In order to confirm the depth of anesthesia, we monitored HR fluctuations (Supplementary Fig. S5). Additionally, we have confirmed whether an alternative ketamine-xylazine anesthesia (ketamine (100mg/kg) and xylazine (10mg/kg) cocktail, IP) produced results comparable to those obtained from the isoflurane anesthesia (Supplementary Fig. S6). All animals (C57BL/6; n = 5 animals) used to investigate anesthetic effects underwent the same care and surgery protocol, and 2 weeks restraint stress regime. During imaging, the heart rate was monitored using a pulse oximeter (PhysioSuite, Kent Scientific, Torrington, USA) to ensure the stable depth of anesthesia.

RBC velocity measurement

We measured the RBC velocity through a line scan. As shown in Supplementary Fig. S8 (A), the capillaries with an analytical length and clearly visible shadows were selected. Line stack images were acquired at a temporal resolution of 0.25 ms for 5 minutes in the selected line. After 2 weeks of stress regime, a line scan

was performed under the same protocol in the same area. A total of 45 ROIs were determined in one acquired line stack, and the RBC velocity was measured in the ROI.

Supplementary figures, video, and tables

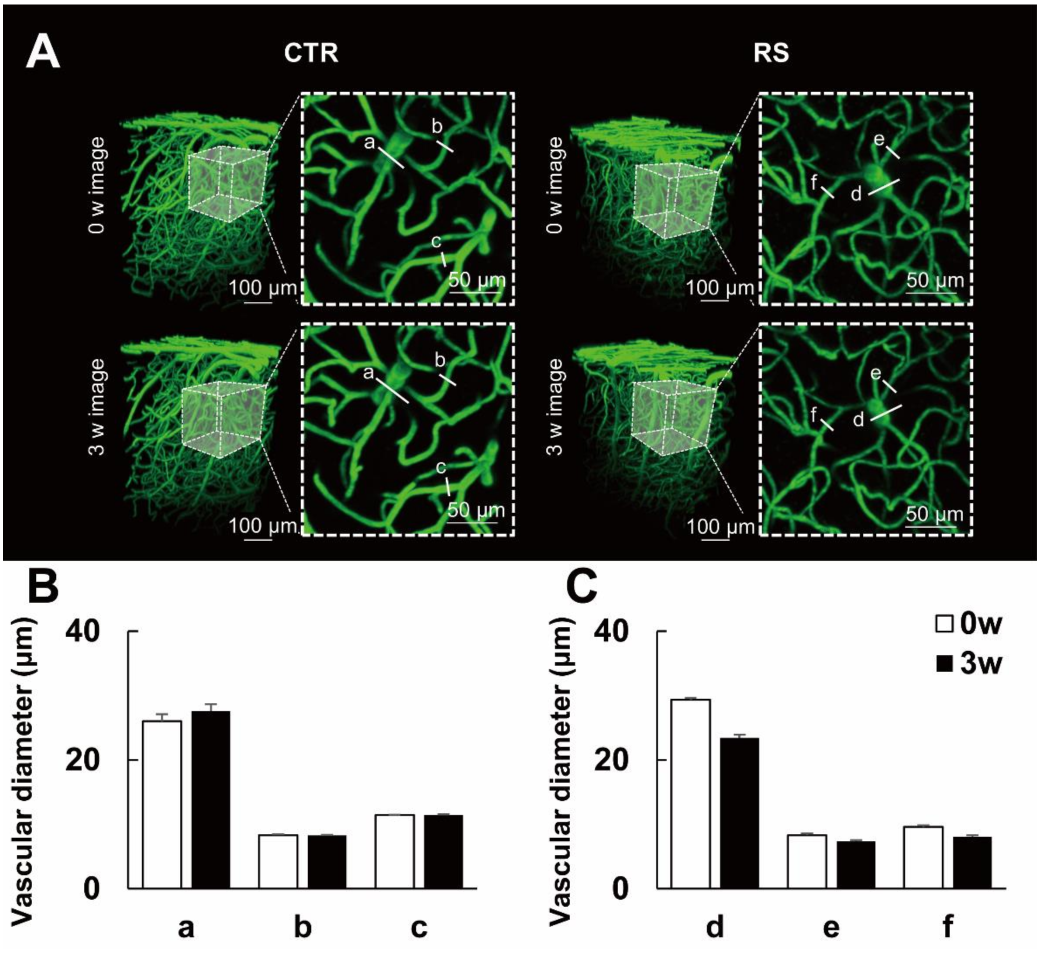

Supplementary Figure S1. Cerebro-vascular structure of an animal following chronic RS. A) Representative images longitudinally acquired in the CTR and RS. Two-dimensional images (dashed square) are the maximum intensity projections of the 3D images with a 100 μm thickness (dashed cube). a-f indicate vessel diameter. B and C) Estimated vascular diameters of the representative images (CTR: a = 26.00 μm to 27.59 μm (0 w to 3 w), b = 8.31 μm to 8.27 μm, and c = 11.44 μm to 11.44 μm; RS: d = 29.32 μm to 23.45 μm, e = 8.34 μm to 7.38 μm, and f = 9.60 μm to 8.10 μm). CTR, control group; RS, restraint stress group.

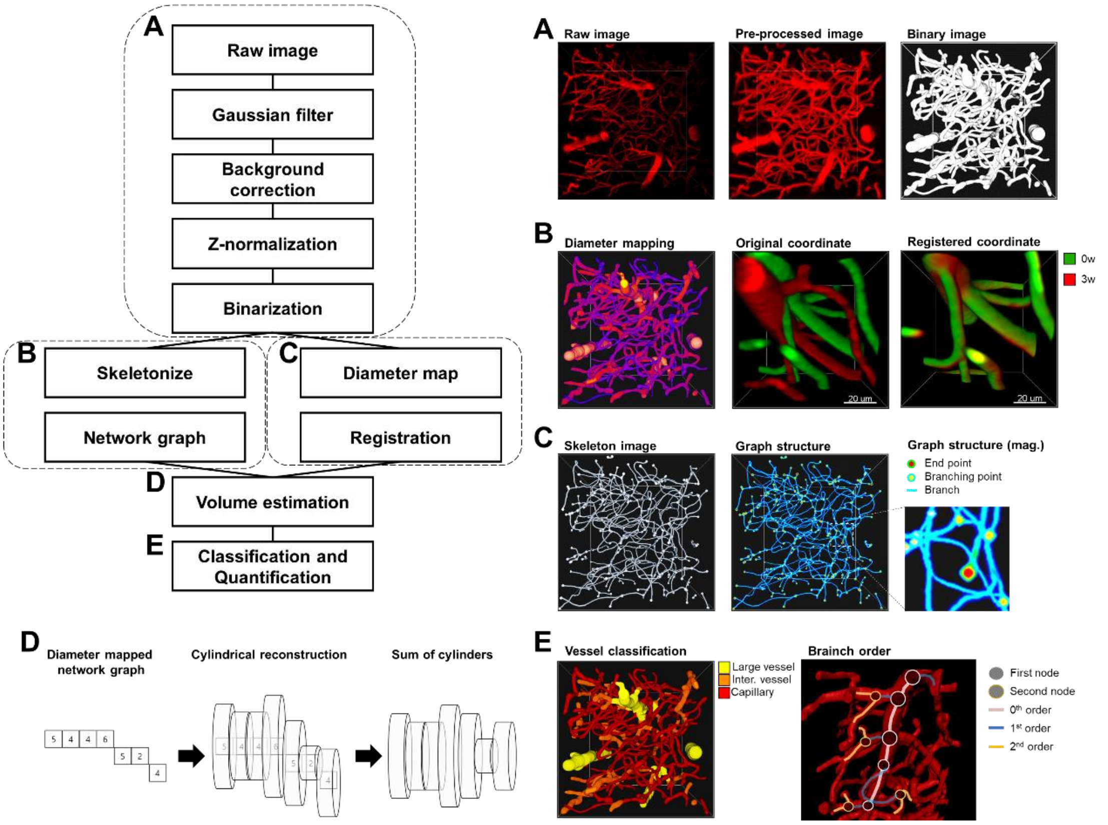

Supplementary Figure S2. Image processing steps to quantify the classified vessels. A) Raw images were pre-processed and binarized using Gaussian filtering, background correction, and z-normalization. B) Diameter map (look up table, fire; Diameter range, 0 μm to 30 μm) and comparisons of the merged longitudinal images between raw and registered coordinates. Green indicates vessels at 0 w, and red indicates vessels at 3 w. C) Skeleton image created from the binary image, and graph structure calculated from the skeleton image. Red points indicate end points, yellow points indicate branching points, and blue lines indicate branches. D) Scheme for reconstructed cerebro-vascular volume. E) Based on the skeleton coordinates and diameter information, all segments were reconstructed using the average diameter. Vascular segment classification was made according to the average diameter. Red indicates capillaries (≤ 9 μm), orange indicates intermediate vessels (≤ 14 μm), and yellow indicates large vessels (> 14 μm). Manually identified pial and penetrating arteries. The starting branch was manually selected first, and the branch with the thickest diameter from the starting branch was identified in order to determine the 0th order vessel. Pink lines indicate the 0th order vessel, blue lines indicate the 1st order vessels and yellow line indicate the 2nd order vessels.

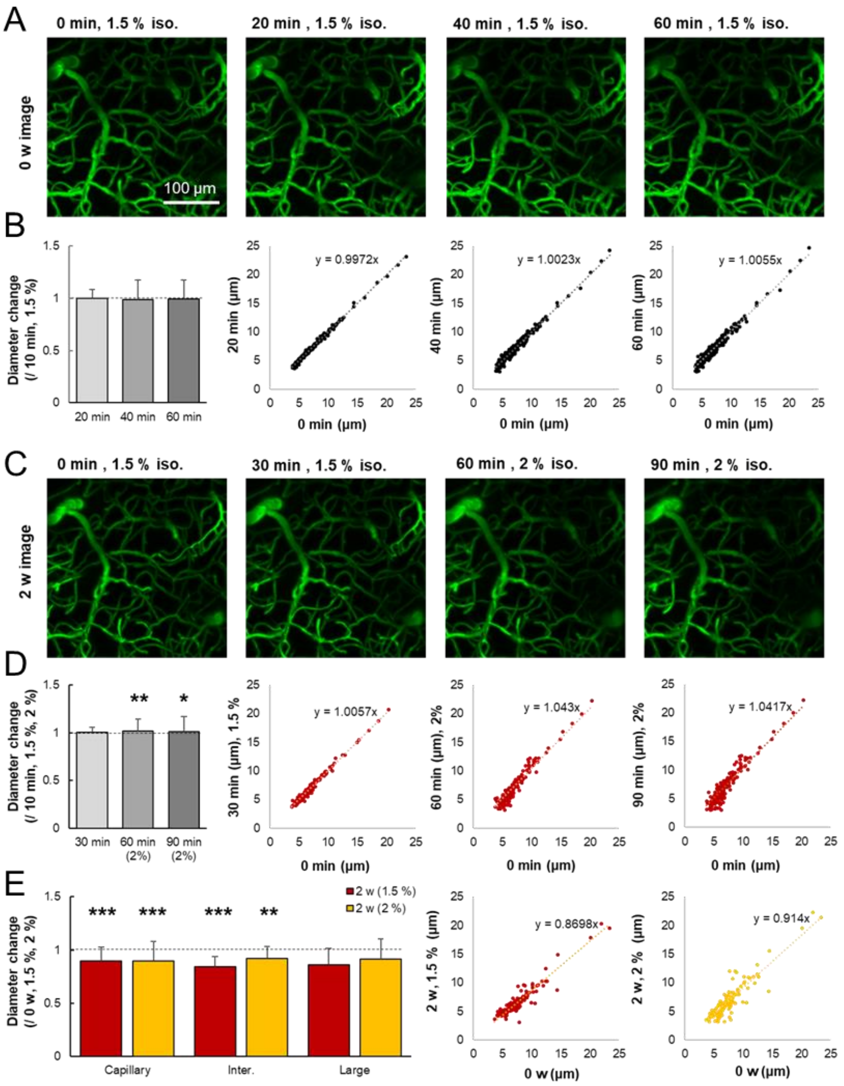

Supplementary Figure S3. A) Series of representative images of baseline (0 w) acquired at 20-minute intervals with 1.5 % isoflurane anesthesia maintained. B) An average value of the vascular diameter change and a scatter plot of the each segment corresponding to (A). The diameter changes at three points were not statistically significant (99.8 ± 8.24 %, 98.9 ± 18.43 % and 99.19 ± 18.32 %). C) A representative images of vessels after stress (2 w) at 30 minute time intervals under 1.5 % and 2 % isoflurane anesthesia, respectively. D) An average value of the vascular diameter change and a scatter plot of the each segment corresponding to (C). 2 w images shows no significant fluctuation of the vessel diameter (0.46 ± 5.62 %) at concentration of 1.5 % isoflurane. Whereas the diameter increased by 1.29 ± 0.54 % at concentration of 2 % isoflurane. E) A capillary, intermediate, and large vessel bar graphs and vessel segments scatter plot of vascular diameter change at 2 w due to stress under different anesthetic concentrations (under 1.5 % isoflurane; capillary, 89.95 ± 12.68 % of 0 w; intermediate, 84.49 ± 9.41 % of 0 w; large, 85.90 ± 15.89 % of 0 w; under 2 % isoflurane; capillary, 89.43 ± 18.70 % of 0 w; intermediate, 91.94 ± 11.60 % of 0 w; large, 91.46 ± 19.29 % of 0 w). The p-value is calculated between 0 w and 2 w in each group by paired t-test. *$p < 0.05$; **$p < 0.01$; ***$p < 0.001$

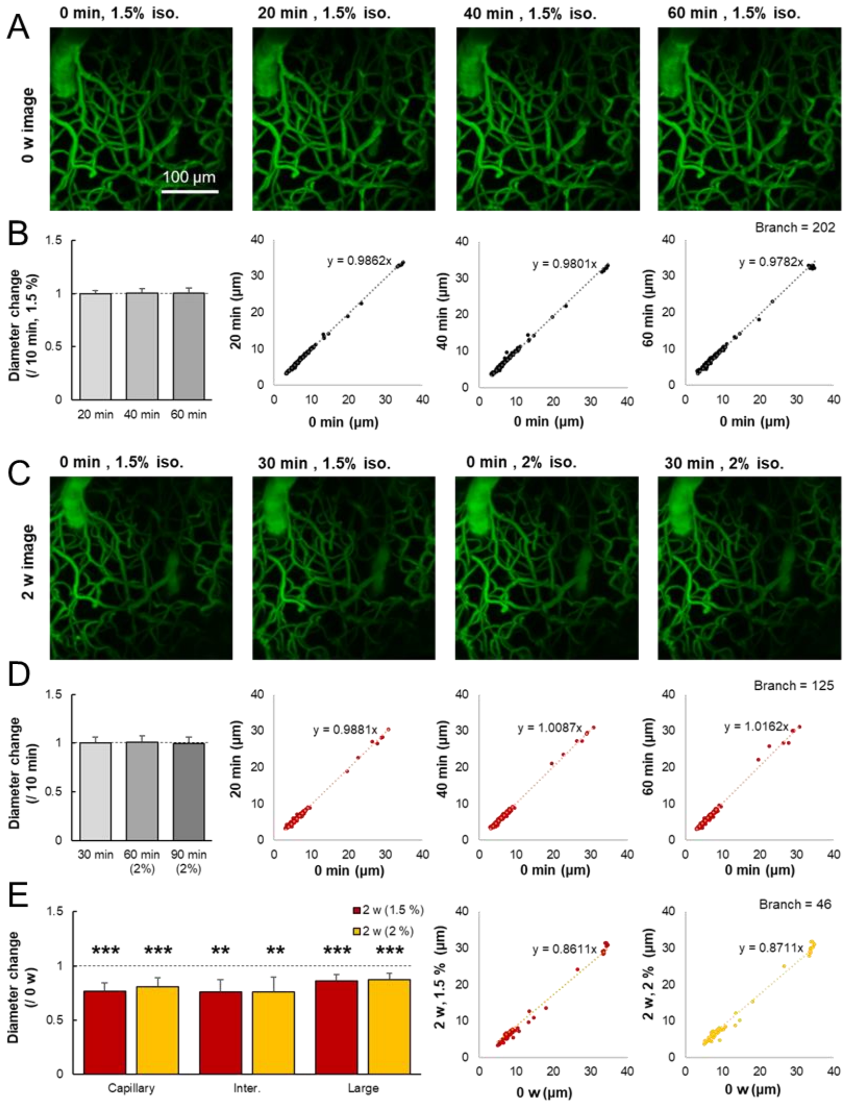

Supplementary Figure S4. A) Series of representative images of baseline (0 w) acquired at 20-minute intervals with 1.5 % isoflurane anesthesia maintained. B) An average value of the vascular diameter change and a scatter plot of the each segment corresponding to (A). The diameter changes at three points were not statistically significant (99.93 ± 2.89 %, 100.33 ± 4.47 % and 100.26 ± 5.06 %). C) A representative images of after stress (2 w) at intervals of 30 minutes under 1.5 % and 2 % isoflurane anesthesia. D) An average value of the vascular diameter change and a scatter plot of the each segment corresponding to (C). 2 w images shows no significant fluctuation of the vessel diameter at concentration of 1.5 % and 2 % (100.54 ± 5.98 %, 101.16 ± 6.28 % and 99.88 ± 6.43 %). E) A capillary, intermediate, and large vessel bar graphs and vessel segments scatter plot of vascular diameter change due to stress under different anesthetic concentrations (under 1.5 % isoflurane; capillary, 76.54 ± 7.89 % of 0 w; intermediate, 76.18 ± 11.19 % of 0 w; large, 86.13 ± 5.75 % of 0 w; under 2 % isoflurane; capillary, 80.7 ± 8.66 % of 0 w; intermediate, 75.74 ± 13.89 % of 0 w; large, 87.19 ± 6.31 % of 0 w). The p-value is calculated between 0 w and 2 w in each group by paired t-test. *$p < 0.05$; **$p < 0.01$; ***$p < 0.001$

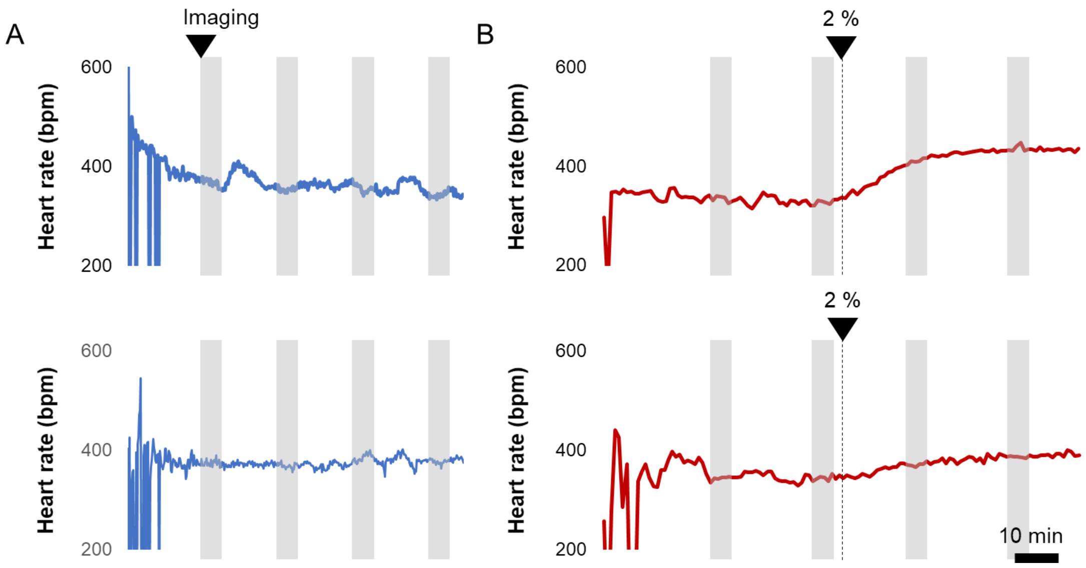

Supplementary Figure S5. HR measurement results for animal #1 and #2. A) The blue line represents the HR change during the 0 w imaging (upper, animal # 1; lower, animal # 2). Imaging was started with stable HR (gray bars indicate imaging time). B) The red line indicates the HR change during imaging at 2 w following stress regime (Upper, animal # 1; below, animal # 2). HR confirmed stable HR as in (A) and began to acquire images. Changes in isoflurane concentration at 2 % showed increased HR in both animals imaging was started after monitoring stable HR.

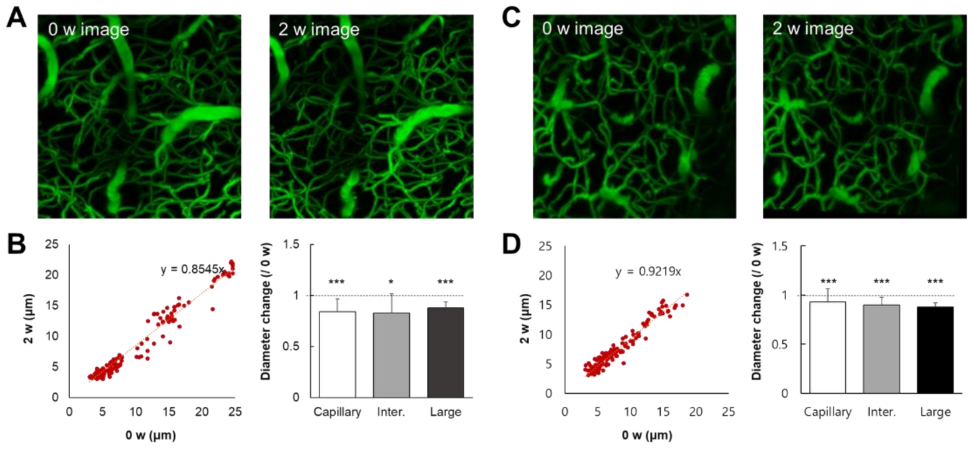

Supplementary Figure S6. Results for alternative ketamine/xylazine anesthesia. A) Representative images of 0 w and 2 w of animal #3. B) An average value of the vascular diameter change according to classification and a scatter plot of the each segment. C) Representative images of 0 w and 2 w of animal #4. D) An average value of the vascular diameter change at 2 w following stress according to classification and a scatter plot of the each segment. (animal #3; capillary, 84.08 ± 12.68 % of 0 w; intermediate, 82.82 ± 18.68 % of 0 w; large, 87.74 ± 5.99 % of 0 w; animal #4; capillary, 92.84 ± 13.74 % of 0 w; intermediate, 90.00 ± 7.64 % of 0 w; large, 87.94 ± 4.03 % of 0 w). The p-value is calculated between 0 w and 2 w in each group by paired t-test. *$p < 0.05$; **$p < 0.01$; ***$p < 0.001$

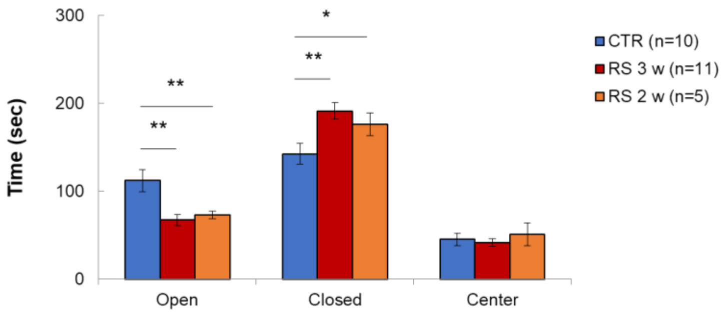

Supplementary Figure S7. Behavioral results of 2 w animals (C57BL/6; n = 5 animals). The p-value is calculated by Kruskal wallis test. *p < 0.05; **p < 0.01; ***p < 0.001

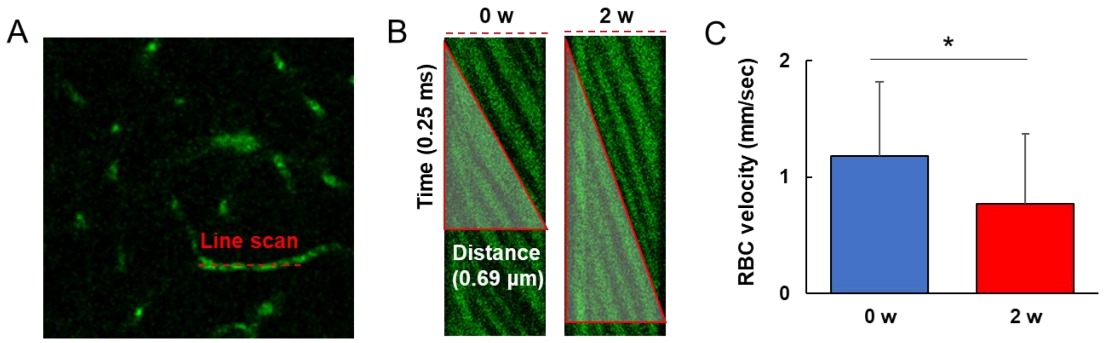

Supplementary Figure S8. Measurement of RBC velocity. A) Representative images of capillaries determined for a line scan. B) Line scan images obtained from the determined capillaries. Left, 0 w; Right, 2 w. ‘Time’ and ‘Distance’ indicate temporal and spatial resolution. C) Mean value of RBC flow obtained from 5 animals (0 w, 1.17 ± 0.64 mm/s; 2 w, 0.77 ± 0.60 mm/s; p = 0.029, paired t-test)

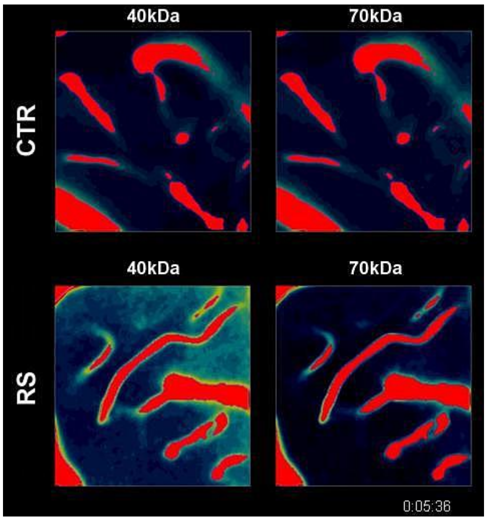

Supplementary Video S1. Representative *in vivo* real time imaging of the extravasation of fluorescence-labeled dextran (40-kDa and 70-kDa). Images were visualized with the color intensity scale. Initial red indicates intra vessel area and initial black indicates extra vessel area. CTR, control group; RS, restraint stress group.

Supplementary Table S1. Comparison of inter-group difference in baseline vessel diameter.

	Number of animal		Capillary (≤9um) 0 w			Intermediate (≤14um) 0 w			Large (≥14um) 0 w		
				Shapiro-Wilk test Sig	t-test Sig.		Shapiro-Wilk test Sig	t-test Sig.		Shapiro-Wilk test Sig	t-test Sig.
CTR	5	average	6.69	0.401	0.950	10.95	0.287	0.622	22.00	0.225	0.517
		s.d.	0.59			0.21			5.46		
RS	6	average	6.71	0.854		10.89	0.762		20.14	0.759	
		s.d.	0.73			0.19			2.78		

Supplementary Table S2. Primer Sequences for real-time PCR

Gene		Sequence	Reference (PMID)
HIF-1α	F	5'-CTC ATC AGT TGC CAC TTC C-3'	25790768
	R	5'-TCA TCT TCA CTG TCT AGA CCA C-3'	
VEGFa	F	5'-GAG AGA GGC CGA AGT CCT TT-3'	21577211
	R	5'-TTG GAA CCG GCA TCT TTA TC-3'	
VEGFR2	F	5'-TGG ACG GAT GAT CAA GAG AA-3'	23122957
	R	5'-CTT CAC AGG GAT TCG GAC TT-3'	
Occludin	F	5'-ATG TCC GGC CGA TGC TCT C-3'	24027765
	R	5'-CTT TGG CTG CTC TTG GGT CTG TAT-3'	
Claudin-3	F	5'-AAG CCG AAT GGA CAA AGA A -3'	23470164
	R	5'-CTG GCA AGT AGC TGC AGT G -3'	
Claudin-5	F	5'-CAA GGT GTA TGA ATC TGT GCT-3'	26409663
	R	5'-GTC AAG GTA ACA AAG AGT GCC A-3'	
beta-actin	F	5'-TGT GAT GGT GGG AAT GGG TCA GAA-3'	22339269
	R	5'-TGT GGT GCC AGA TCT TCT CCA TGT-3'	
